# A full-length glycoprotein mRNA vaccine confers complete protection against severe fever with thrombocytopenia syndrome virus, with broad-spectrum protective effects against bandaviruses

**DOI:** 10.1128/jvi.00769-24

**Published:** 2024-06-03

**Authors:** Jia Lu, Jun Liu, Yan Wu, Xiaoxue He, Xiao Gao, Xinlan Chen, Shaoyi Chen, Xuerui Zhu, Yucai Peng, Gengfu Xiao, Xiaoyan Pan

**Affiliations:** 1 State Key Laboratory of Virology, Wuhan Institute of Virology, Chinese Academy of Sciences, Wuhan, China; 2 University of the Chinese Academy of Sciences, Beijing, China; 3 Liverna Therapeutics Inc., Zhuhai, China; 4 Key Laboratory of Virology and Biosafety, Wuhan Institute of Virology, Chinese Academy of Sciences, Wuhan, China; Lerner Research Institute, Cleveland Clinic, Cleveland, Ohio, USA

**Keywords:** severe fever with thrombocytopenia syndrome virus, glycoprotein, cellular immunology, broad-spectrum protection, conserved epitope

## Abstract

Highly pathogenic viruses from family *Phenuiviridae*, which are mainly transmitted by arthropods, have intermittently sparked epidemics worldwide. In particular, tick-borne bandaviruses, such as severe fever with thrombocytopenia syndrome virus (SFTSV), continue to spread in mountainous areas, resulting in an average mortality rate as high as 10.5%, highlighting the urgency and importance of vaccine development. Here, an mRNA vaccine developed based on the full-length SFTSV glycoprotein, containing both the receptor-binding domain and the fusion domain, was shown to confer complete protection against SFTSV at a very low dose by triggering a type 1 helper T cell-biased cellular immune response in rodents. Moreover, the vaccine candidate elicited long-term immunity and protection against SFTSV for at least 5 months. Notably, it provided complete cross-protection against other bandaviruses, such as the Heartland virus and Guertu virus, in lethal challenge models. Further research revealed that the conserved epitopes among bandaviruses within the full-length SFTSV glycoprotein may facilitate broad-spectrum protection mediated by the cellular immune response. Collectively, these findings demonstrate that the full-length SFTSV glycoprotein mRNA vaccine is a promising vaccine candidate for SFTSV and other bandaviruses, and provide guidance for the development of broad-spectrum vaccines from conserved antigens and epitopes.

**IMPORTANCE:**

Tick-borne bandaviruses, such as SFTSV and Heartland virus, sporadically trigger outbreaks in addition to influenza viruses and coronaviruses, yet there are no specific vaccines or therapeutics against them. mRNA vaccine technology has advantages in terms of enabling *in situ* expression and triggering cellular immunity, thus offering new solutions for vaccine development against intractable viruses, such as bandaviruses. In this study, we developed a novel vaccine candidate for SFTSV by employing mRNA vaccination technology and using a full-length glycoprotein as an antigen target. This candidate vaccine confers complete and durable protection against SFTSV at a notably low dose while also providing cross-protection against Heartland virus and Guertu virus. This study highlights the prospective value of full-length SFTSV-glycoprotein-based mRNA vaccines and suggests a potential strategy for broad-spectrum bandavirus vaccines.

## INTRODUCTION

Ticks, mosquitoes, and sandflies are ubiquitous vectors that facilitate the global spread of viruses and occasionally transmit arboviruses from domesticated animals and poultry to humans (1–3). These arboviruses, including flaviviruses, togaviruses, and bunyaviruses, cause various serious infectious diseases and even death (4, 5). Among them, the tick-borne severe fever with thrombocytopenia syndrome virus (SFTSV) and Heartland virus (HRTV), belonging to the genus *Bandavirus*, family *Phenuiviridae*, and order *Bunyavirales*, cause similar severe clinical manifestations, including fever, thrombocytopenia, leukopenia, multisystem organ failure, and even death (6, 7). Specifically, SFTSV has an average case-fatality rate of 10.5%, and HRTV has a mortality rate ranging from 13% to 30% (8–11). In addition, an increasing number of tick-borne bandaviruses, such as Guertu virus (GTV), which was discovered in Xinjiang, China, have been found to be potentially pathogenic to humans (12). Viruses other than bandaviruses in the family *Phenuiviridae*, including the tick-borne Khasan virus (KHAV) (13) and Razdan virus (RAZV) (14), the mosquito-transmitted Rift Valley fever virus (RVFV) (15), and the sandfly-transmitted sandfly fever Sicilian virus (SFSV) (16), also cause sporadic deaths worldwide. Together, viruses constitute a global public health threat, in addition to respiratory viruses, such as influenza viruses and coronaviruses, highlighting the urgent need to develop specific prevention and treatment strategies (4).

Classical bandaviruses are enveloped and segmented, negative-stranded RNA viruses. Their genomes consist of small (S), medium (M), and large (L) segments encoding the nucleoprotein (NP), non-structural protein (NS), envelope glycoprotein (GP), and large polymerase protein (LP), respectively (17). Using SFTSV as an example to illustrate the life cycle of bandaviruses, the M segment expresses a glycoprotein precursor within the endoplasmic reticulum, which is then cleaved into the Gn and Gc subunits that subsequently form heterodimers and are immediately incorporated into virions as pentons and hexons within the Golgi apparatus (18). Next, mature virions are transported to the plasma membrane via vesicles, culminating in the release and completion of the viral replication cycle (19). When SFTSV enters a host cell, Gn initiates an infection by interacting with dendritic cell-specific intercellular adhesion molecule 3-grabbing non-integrin and C-C motif chemokine receptor 2 to implement binding (20–22), which is accompanied by membrane fusion induced by Gc within acidic endosomes (23). Given the essential roles of Gn and Gc in the viral life cycle, GPs containing both Gn and Gc are regarded as attractive targets for therapeutic antibodies and vaccines (24–27).

Because of the complex conformation of the SFTSV GP, which is difficult to reproduce *in vitro*, previously reported vaccines based on recombinant Gn or Gc proteins have not provided satisfactory protective effects (28). However, mRNA technology overcomes these intricacies by allowing the direct expression of antigens *in situ*, eliminating much of the uncertainty associated with traditional vaccine preparation (29). The efficacy and safety of mRNA vaccines have been substantiated through extensive real-world evidence, as exemplified during the coronavirus disease 2019 pandemic (30, 31), thereby affirming their potential for application against a broader range of pathogens. In this study, an mRNA vaccine was constructed based on the full-length SFTSV GP, and its immunogenicity and efficacy were investigated. The vaccine provided complete protection against SFTSV and broad-spectrum protection against other bandaviruses. The cellular immune response, which may be partially engaged by the conserved epitopes, was deemed to be the main mechanism. This study principally focused on the efficacy of a full-length *GP* mRNA vaccine candidate and its associated mechanisms to propose a viable approach for developing broad-spectrum vaccines.

## RESULTS

### The *GP* mRNA vaccine elicited humoral and type 1 helper T cell-biased cellular immune responses in mice

**Fig 1:**
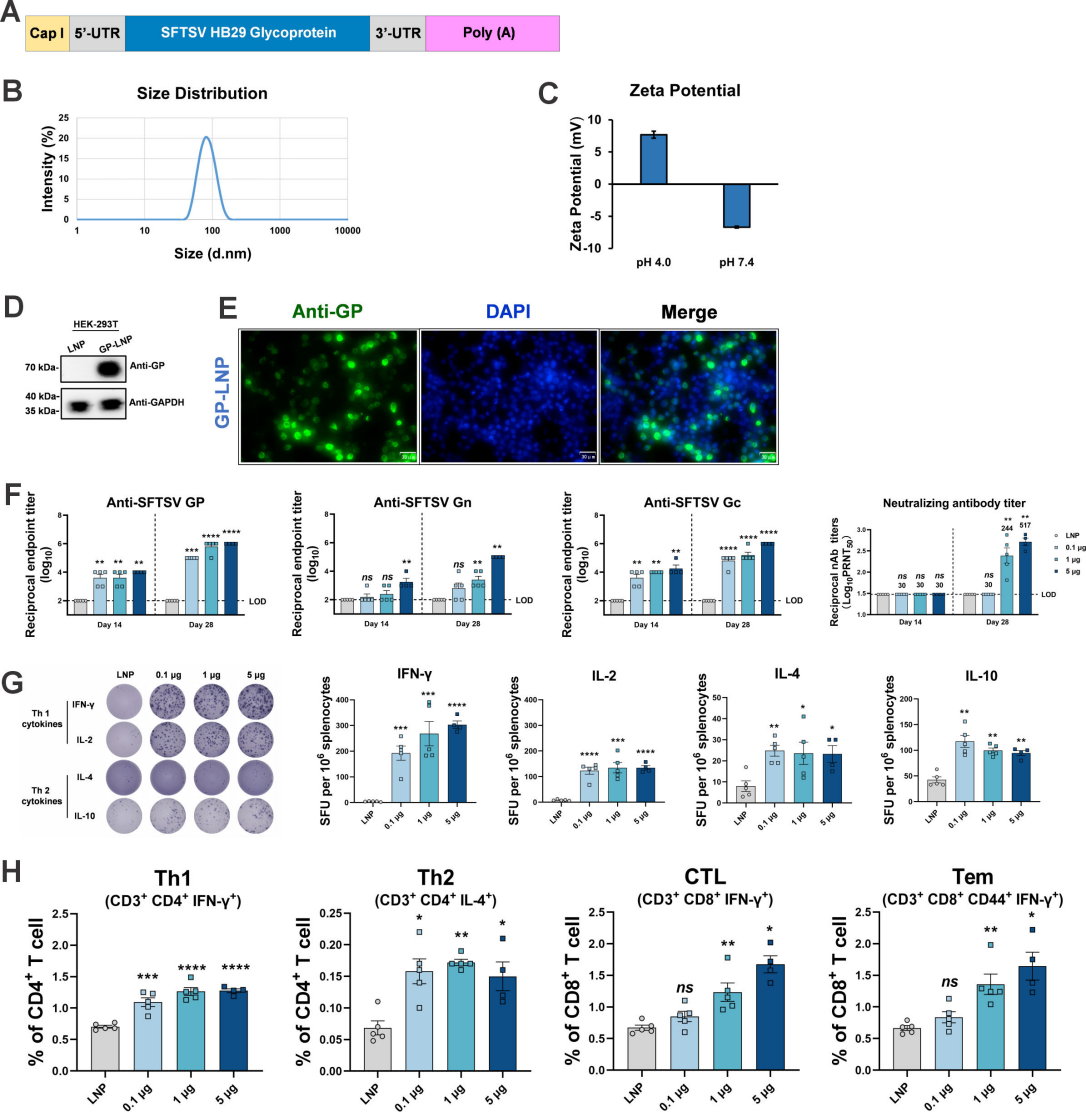
Immunogenicity evaluation of the *GP* mRNA vaccine in BALB/c mice. (A) Schematic diagram of the full-length SFTSV *GP* mRNA vaccine. (B and C) Size distribution and zeta potential of the mRNA-LNPs. (D and E) The expression of SFTSV GP mRNA in HEK-293T cells was detected by Western blotting with glyceraldehyde-phosphate dehydrogenase used as the control (also shown in Fig. **S2**), and visualized in the cytoplasm using immunofluorescence with nuclei stained with 4′,6-diamidino-2-phenylindole. (F–H) Female BALB/c mice were allocated into the following four groups with five per group: empty LNPs, 0.1, 1.0, or 5.0 μg of GP-LNPs. Vaccinations followed a standard prime-boost regimen with a 3-week interval. The levels of binding antibodies against SFTSV GP, Gn, and Gc were determined by enzyme-linked immunosorbent assay with a starting dilution of 1:100 (LOD). Neutralizing antibodies were ascertained using a plaque reduction neutralization test, with geometric mean titers indicated above the corresponding bars (LOD of 1:30) (F). The cellular immune response was detected by either ELISpot or flow cytometry. Representative images (left panel) and spots of IFN-γ, IL-2, IL-4, and IL-10 (right panel) are shown (G), and the proportions of Th1 cells, Th2 cells, CTLs, and Tem cells were analyzed via ICS assay (H). *P < 0.05, **P < 0.01, ***P < 0.001, ****P < 0.0001. CTL, cytotoxic T cell; DAPI, 4′,6-diamidino-2-phenylindole; ELISpot, enzyme-linked immunospot assay; ICS, intracellular cytokine staining assay; GP, glycoprotein; IFN-γ, interferon gamma; IL, interleukin; LNP, lipid nanoparticle; LOD, limit of detection; ns, not significant; SFTSV, severe fever with thrombocytopenia syndrome virus; Tem, effector memory T; Th1, type 1 helper T; Th2, type 2 helper T; UTR, untranslated region.

For vaccine preparation, a template containing codon-optimized full-length SFTSV GP was *de novo* synthesized and then *in vitro* transcribed into mRNA, with uridines substituted by N1-methyl-pseudouridines (Fig. 1A). Microchip capillary electrophoresis showed that the *GP* mRNA had a length of 3,490 nt, which is consistent with the theoretical size of the transcript, and a purity of 98.3% (Fig. S1). Subsequently, the mRNA was encapsulated within lipid nanoparticles (LNPs), which were formulated with a specific ratio of ionizable cationic lipids, phosphatidylcholine, cholesterol, and polyethylene glycol lipids, to produce GP-LNPs. Dynamic light scattering showed that the GP-LNPs had a particle size of approximately 80 nm (Fig. 1B), and the zeta potentials at pH 4.0 and 7.4 were 7.69 and −6.70, respectively (Fig. 1C), suggesting its appropriate uniformity and stability. Successful expression in mammalian cells was confirmed in HEK-293T cells by Western blotting and immunofluorescence (Fig. 1D and E; Fig. S2).

To investigate the immunogenicity of the *GP* mRNA vaccine, immunocompetent BALB/c mice were intramuscularly (i.m.) vaccinated with various doses of GP-LNPs ranging from 0.1 to 5.0 µg. After receiving a two-shot vaccination regimen with a 3-week interval and biweekly sampling, the mice were sacrificed to analyze both the humoral and cellular immune responses 10 days after the booster vaccination. As expected, the GP-LNPs elicited high specific binding titers against SFTSV GP, Gn, and Gc, which peaked at ~1:10^6^ after booster vaccination with 5 µg of GP-LNPs, in a dose-dependent manner. Additionally, the neutralizing antibody titers exhibited the same trend as the binding antibody titers, reaching a reciprocal geometric mean titer (GMT) of 1:517 after booster vaccination with 5 µg of GP-LNPs, despite the absence of significant neutralizing antibodies after priming vaccination (Fig. 1F).

The cellular immune response assessed by enzyme-linked immunospot assay (ELISpot) showed robust type 1 helper T (Th1) cytokines [interferon gamma (IFN-γ) and interleukin (IL)-2] production by the SFTSV GP peptide pool stimulation even at a vaccination dose as low as 0.1 µg, compared with the relatively weak Th2-type cytokine (IL-4 and IL-10) production (Fig. 1G). Consistent with these results, the proportions of Th1 cells (CD3^+^, CD4^+^, and IFN-γ^+^), cytotoxic T cells (CD3^+^, CD8^+^, and IFN-γ^+^), and effector memory T cells (CD3^+^, CD8^+^, CD44^+^, and IFN-γ^+^) detected by flow cytometry were dose-dependently elevated with the vaccination doses after stimulation with the SFTSV GP peptide pool, while those of Th2 cells (CD3^+^, CD4^+^, and IL-4^+^) remained at a low level despite having statistical significance (Fig. 1H; Fig. S3). Collectively, these results demonstrated that both humoral and Th1-biased cellular immune responses can be elicited by the SFTSV *GP* mRNA vaccine, enabling a subsequent efficacy evaluation.

### The *GP* mRNA vaccine conferred complete protection against lethal SFTSV challenge at a low dose

**Fig 2:**
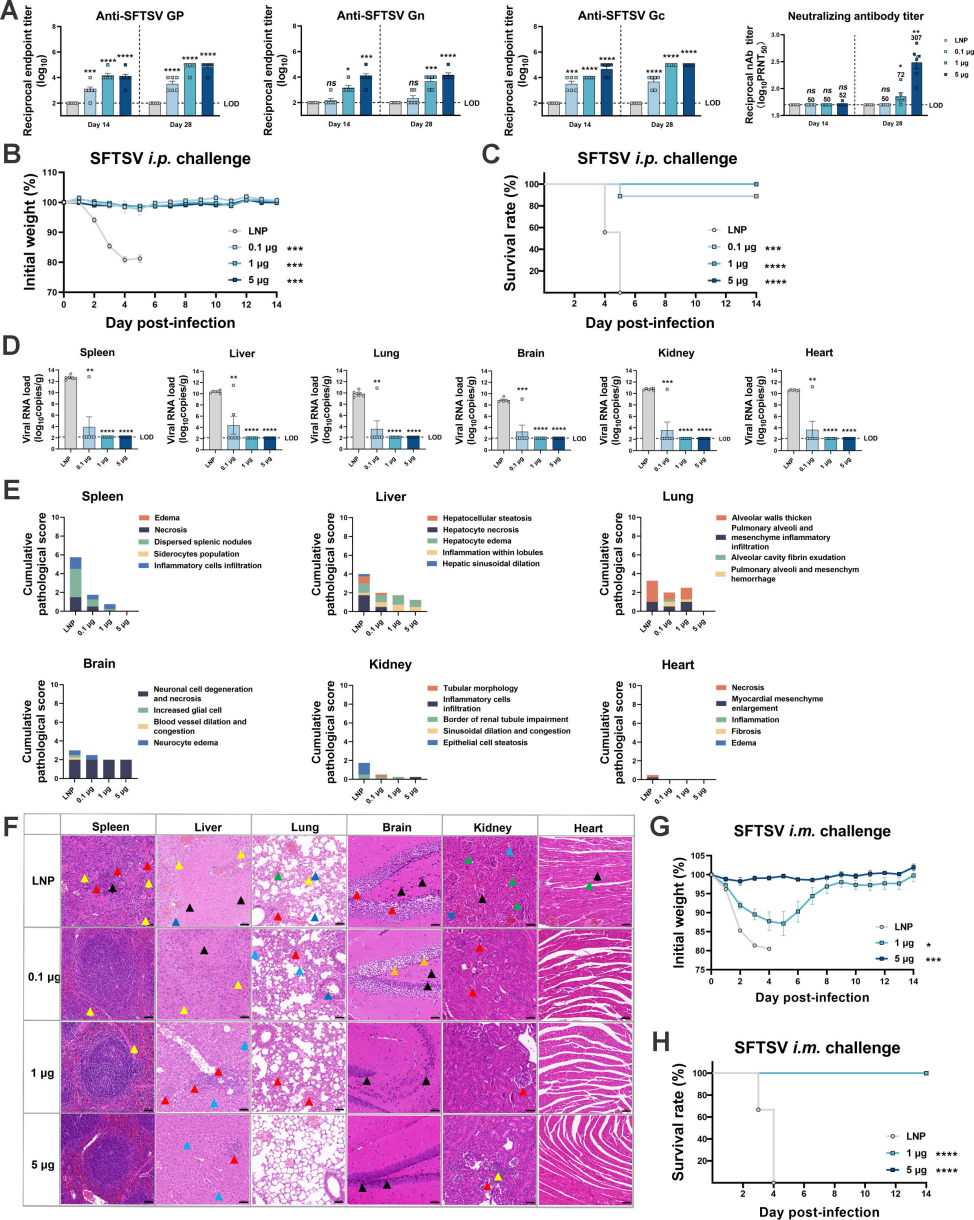
Dose-dependent efficacy of the *GP* mRNA vaccine in A129 mice. A129 mice received vaccinations with empty LNPs, 0.1, 1.0, or 5.0 µg of GP-LNPs with a regular two-shot regimen, followed by i.p. challenge with 100 TCID_50_ of SFTSV 10 days after the booster vaccination. (A) Binding and neutralizing antibodies were detected using ELISAs for GP, Gn, or Gc and PRNT, respectively. The LODs for the ELISA and PRNT were 1:100 and 1:50, respectively. (B and C) Body weight and survival were monitored over 14 days post-challenge. (D) The viral loads in the spleen, liver, lung, brain, kidney, and heart were detected by reverse transcription-quantitative polymerase chain reaction based on the standard curve method with a pair of primers targeting the *NP* gene. The LOD for qPCR was 138 copies per reaction, and converted copies are presented. (E) Cumulative pathological scores for different tissues from each mouse were calculated based on pathological indicators; the details of the scoring criteria are provided in the supplemental materials. (F) Representative images from each group were analyzed by hematoxylin and eosin staining. The colored arrows in the images marking obvious pathological features and their detailed meanings are listed as follows: (i) lungs: red indicates alveolar epithelial cells proliferated, alveolar atrophy, and alveolar walls thickened; green denotes congested and dilated capillaries within the alveolar wall; blue indicates protein mucus present in the bronchus lumen; yellow denotes inflammatory cell infiltration; (ii) liver: red indicates hepatocellular necrosis, nuclear fragmentation, and karyopyknosis; green denotes hepatocyte steatosis; black indicates hepatic sinusoidal dilatation; blue denotes hepatocyte edema; yellow indicates inflammatory cell infiltration; (iii) kidneys: red denotes glomerular atrophy and decreased number of mesangial cells; green indicates glomeruli were lobulated or had interstitial hemorrhages; black denotes degeneration of renal tubular epithelial cells; blue denotes protein mucus present in the kidney tubules; yellow denotes inflammatory cell infiltration; (iv) spleen: red denotes necrosis, nuclear fragmentation, and karyopyknosis of lymphocytes; black indicates scattered splenic nodules and blurred boundary between the red pulp; yellow denotes inflammatory cell infiltration; (v) brain: red indicates neuronal cell degeneration, karyopyknosis, and basophilia were enhanced; blue denotes neuronal cell edema; yellow denotes increased number of glial cells; green indicates congested and dilated blood vessels; (vi) heart: black denotes myocardial fibrosis and rupture; blue: indicates disordered arrangement of myocardial fibers and connective tissue hyperplasia; yellow denotes inflammatory cell infiltration. (G and H) A129 mice received vaccinations with empty LNPs, 1 or 5 µg of GP-LNPs with a regular two-shot regimen, followed by i.m. challenge with 1 × 10^5^ TCID_50_ of SFTSV 10 days after the booster vaccination. Body weight and survival were monitored for 14 days. ELISA, enzyme-linked immunosorbent assay; i.m., intramuscular; i.p., intraperitoneal; PRNT, plaque reduction neutralization test; qPCR, quantitative PCR; TCID_50_, 50% tissue culture infectious dose.

The efficacy of the *GP* mRNA vaccine was evaluated in a type I interferon receptor-deficient (IFNαR^−/−^) C57BL/6 J mouse (A129) model, with the same vaccination regimen used in the immunogenicity investigation. After the vaccination, A129 mice were intraperitoneally (i.p.) challenged with 100, 50% tissue culture infectious dose (TCID_50_) of SFTSV, followed by monitoring the body weight and survival rate for 2 weeks. As shown in Fig. 2A, the antibody responses, including binding antibodies against GP, Gn, and Gc, and neutralizing antibodies against SFTSV, in A129 mice were consistent with those in BALB/c mice, and a dose-dependent effect was seen. It should be noted that significant neutralization occurred at a dose of 5 µg, while a dose less than 1 µg elicited negligible neutralizing antibodies.

After the challenge, vehicle-treated mice experienced sustained body weight loss and died within 5 days post-infection (dpi). In contrast, mice vaccinated with 1- and 5-µg GP-LNPs maintained their body weights and no deaths occurred within 14 days, whereas one mouse vaccinated with 0.1 µg of GP-LNPs died at day 5 (one of nine) (Fig. 2B and C). At the experimental endpoint, the spleens, livers, lungs, brains, kidneys, and hearts were dissected to examine viral loads and pathological lesions. Compared to those in the vehicle-treated group (viral loads as high as 10^10^ copies/g), the viral loads in these tissues of surviving mice were effectively reduced by the *GP* mRNA vaccine in a dose-dependent manner (Fig. 2D). Consistent with the viral loads, pathological injuries to these tissues were eliminated or alleviated by the *GP* mRNA vaccine (Fig. 2E and F).

To evaluate the efficacy of the *GP* mRNA vaccine under a more realistic infectious route, another set of SFTSV challenge experiments was conducted by i.m. inoculating A129 mice with 1 × 10^5^ TCID_50_ of SFTSV. Unlike the i.p. challenge, vehicle-treated mice experienced more rapid weight loss and died within 4 dpi. Although the body weight showed more dramatic fluctuations in the 1-µg than the 5-µg group, all of the mice survived (Fig. 2G and H). These data indicated the explicit protection provided by the *GP* mRNA vaccine against SFTSV at a low dose under different infection conditions.

### A two-shot regimen was required for complete protection by a low dose of the *GP* mRNA vaccine

**Fig 3:**
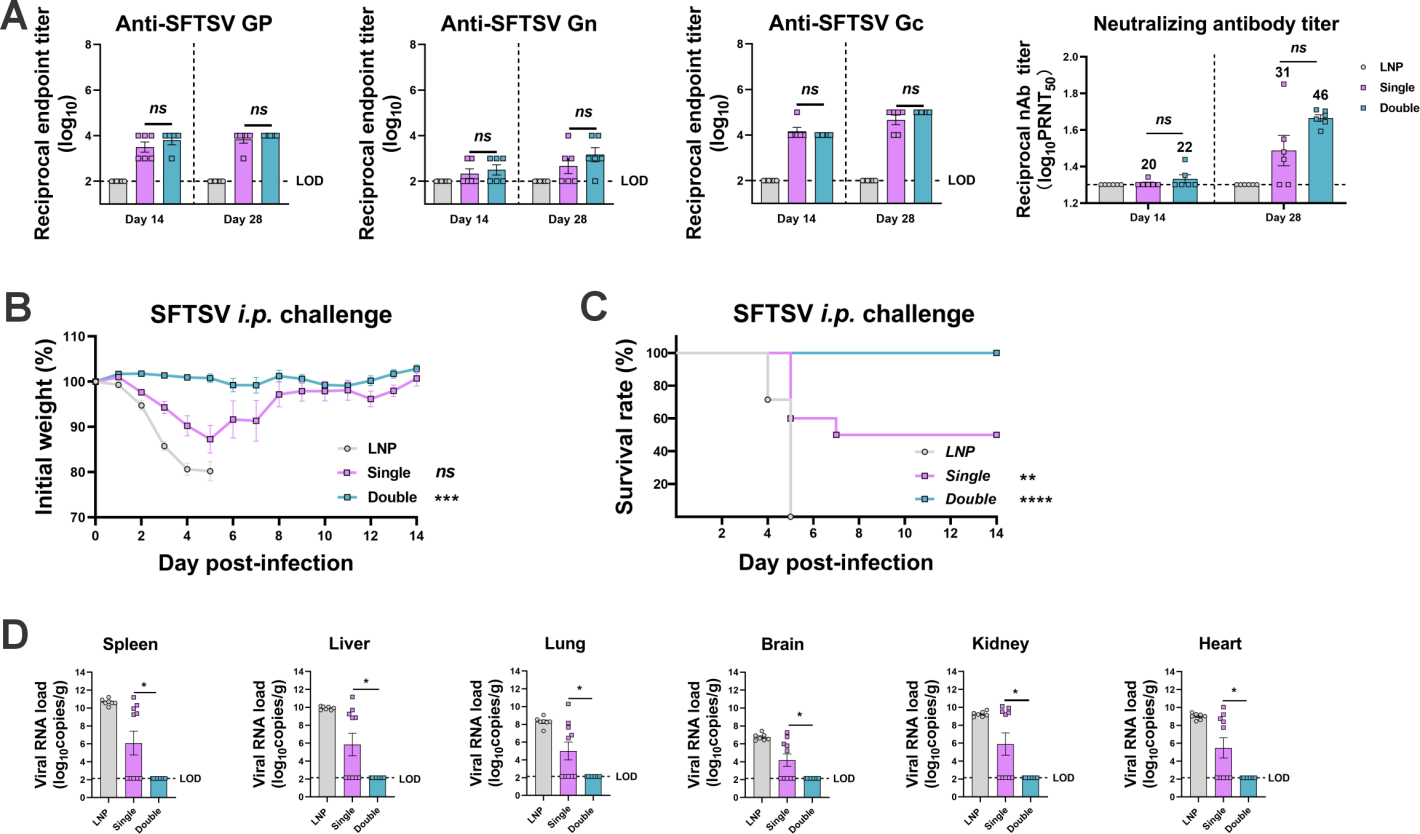
Efficacy of the *GP* mRNA vaccine in single-dose and double-dose regimens. A129 mice received vaccinations with empty LNPs or 1 µg of GP-LNPs in a single- or double-dose regimen, followed by i.p. challenge with 100 TCID_50_ of SFTSV. (A) Binding antibodies against SFTSV GP, Gn, and Gc were detected by ELISA (LOD, 1:100). Neutralizing antibodies were detected by PRNT, with the GMTs indicated above the corresponding bars (LOD, 1:20). (B and C) Curves of body weight and survival, which were monitored for 14 days post-challenge. (D) Viral loads in the spleen, liver, lung, brain, kidney, and heart were detected by reverse transcription-quantitative PCR (LOD, 138 copies). *P < 0.05, **P < 0.01, ***P < 0.001, ****P < 0.0001.

Next, we fixed the dose at 1 µg and changed the vaccination frequency to preliminarily investigate the vaccination procedure. From the antibody results, it can be concluded that the GP-LNPs triggered limited production of binding or neutralization antibodies, regardless of whether a single- or double-dose regimen was applied (Fig. 3A). According to the challenge data, only 50% of the mice (5 of 10) in the single-dose group survived, whereas 100% of the mice in the double-dose group survived (Fig. 3B and C). As shown in Fig. 3D, the double-dose regimen significantly reduced the viral loads in tissues such as the spleen, liver, lung, brain, kidney, and heart, whereas the single-dose regimen alone did not, which is consistent with the body weight and survival rate data. These results further confirm that a booster vaccination is necessary for the *GP* mRNA vaccine to give rodents complete protection, particularly when administered at a relatively low dose, such as 1 µg. Moreover, the data suggested that the protective effect can manifest without a strong humoral immune response, which emphasizes the role of the cellular immune response in the protection offered by this *GP* mRNA vaccine.

### The SFTSV *GP* mRNA vaccine conferred long-term protection against lethal challenge

**Fig 4:**
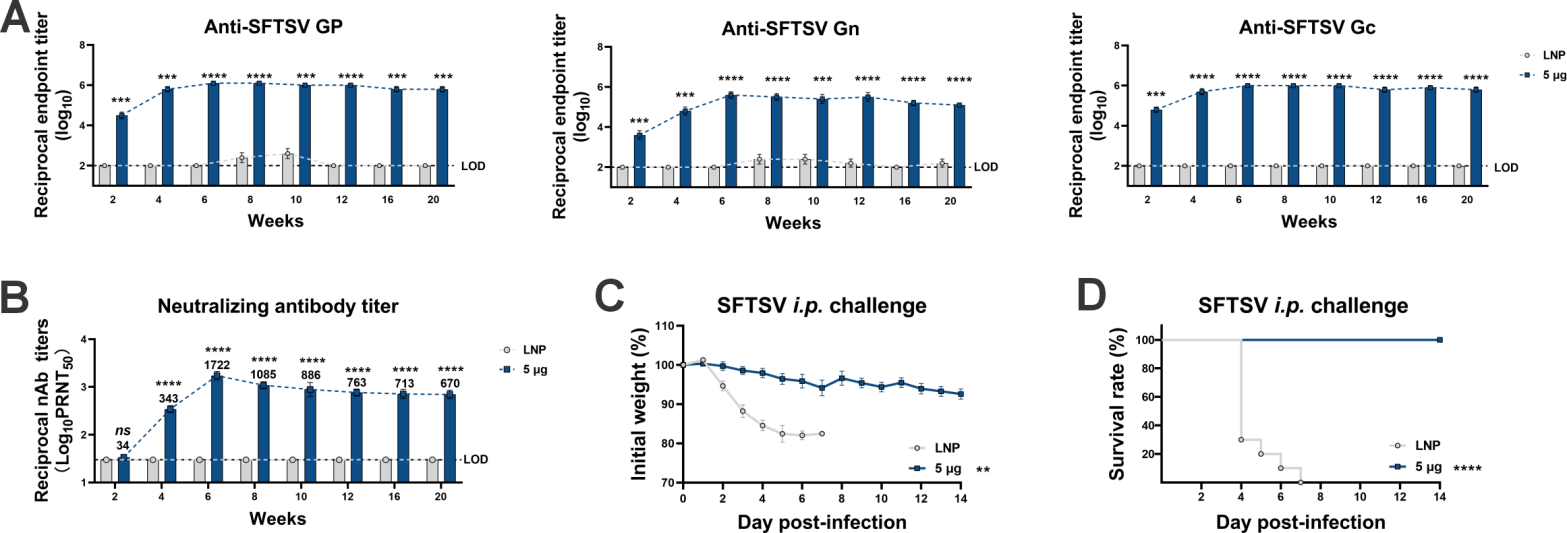
Long-term immune response and protection provided by the *GP* mRNA vaccine. A129 mice were vaccinated with empty LNPs or 5 µg of GP-LNPs following a two-shot regimen and were then were challenged with 100 TCID_50_ of SFTSV (i.p.) 21 weeks later. (A) The titers of binding antibodies specific to SFTSV GP, Gn, or Gc lasting for 20 weeks were detected by ELISA (LOD, 1:100). (B) The neutralizing antibody titers within 20 weeks were detected by PRNT (LOD, 1:30). (C and D) The body weight and survival rate were monitored for 14 days post-challenge. **P < 0.01, ***P < 0.001, ****P < 0.0001.

Encouraging outcomes associated with the *GP* mRNA vaccine prompted us to investigate the effective protection period. Although 1 µg was confirmed to be the optimal dose, 5 µg was used in this study to trigger a more robust humoral response to be convenient for immune indicator detection. Following the standard two-dose regimen, the mice were subjected to biweekly sampling for 20 weeks (approximately 5 months) and were then challenged in the 21st week. The results showed that the peak titers of GP-, Gn-, and Gc-specific binding antibodies all occurred in week 6 and subsequently stabilized at approximately 1:10^6^ or experienced only a marginal decline over the 20-week span. Similarly, the neutralizing antibody titers peaked in week 6 and then decreased slightly but remained high until week 20 (Fig. 4A and B). Upon challenge, it was determined that complete protection was achieved, as reflected by the maintenance of body weight and 100% survival rate over 14 days (Fig. 4C and D). These results demonstrated the long-term immune response and protection offered by the *GP* mRNA vaccine, further indicating its efficacy.

### The *GP* mRNA vaccine provided cross-protection against other viruses from the genus *Bandavirus*, including Heartland virus and Guertu virus

**Fig 5:**
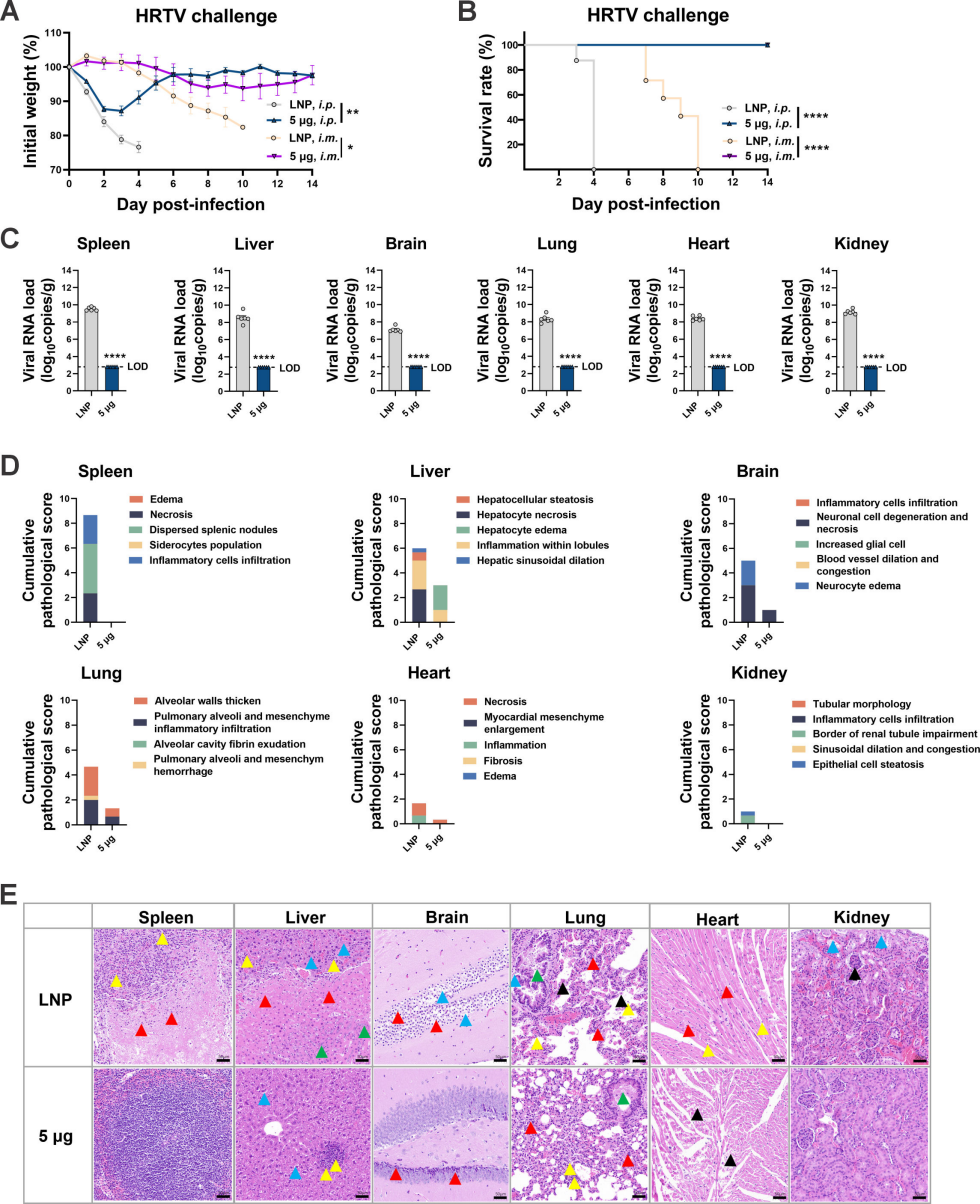
Cross-protection offered by the *GP* mRNA vaccine against Heartland virus. A129 or AG129 mice were vaccinated with empty LNPs or 5 µg of GP-LNPs in a two-shot regimen and were subsequently challenged with HRTV (A129, i.p., 1 × 10^7^ TCID_50_; AG129, i.m., 1 × 10^5^ TCID_50_). (A and B) Body weight and survival were monitored for 14 days post-challenge. (C) viral loads in the tissues after i.p. challenge were quantified using reverse transcription-quantitative PCR, as described above (LOD, 629 copies). (D and E) Cumulative pathological scores and representative images from each tissue are presented. A detailed description of the pathological analysis is listed in the supplemental materials, and annotations for the colored arrows can be referred to in the Fig. 2 legend. *P < 0.05, **P < 0.01, ****P < 0.0001. HRTV, Heartland virus.

**Fig 6:**
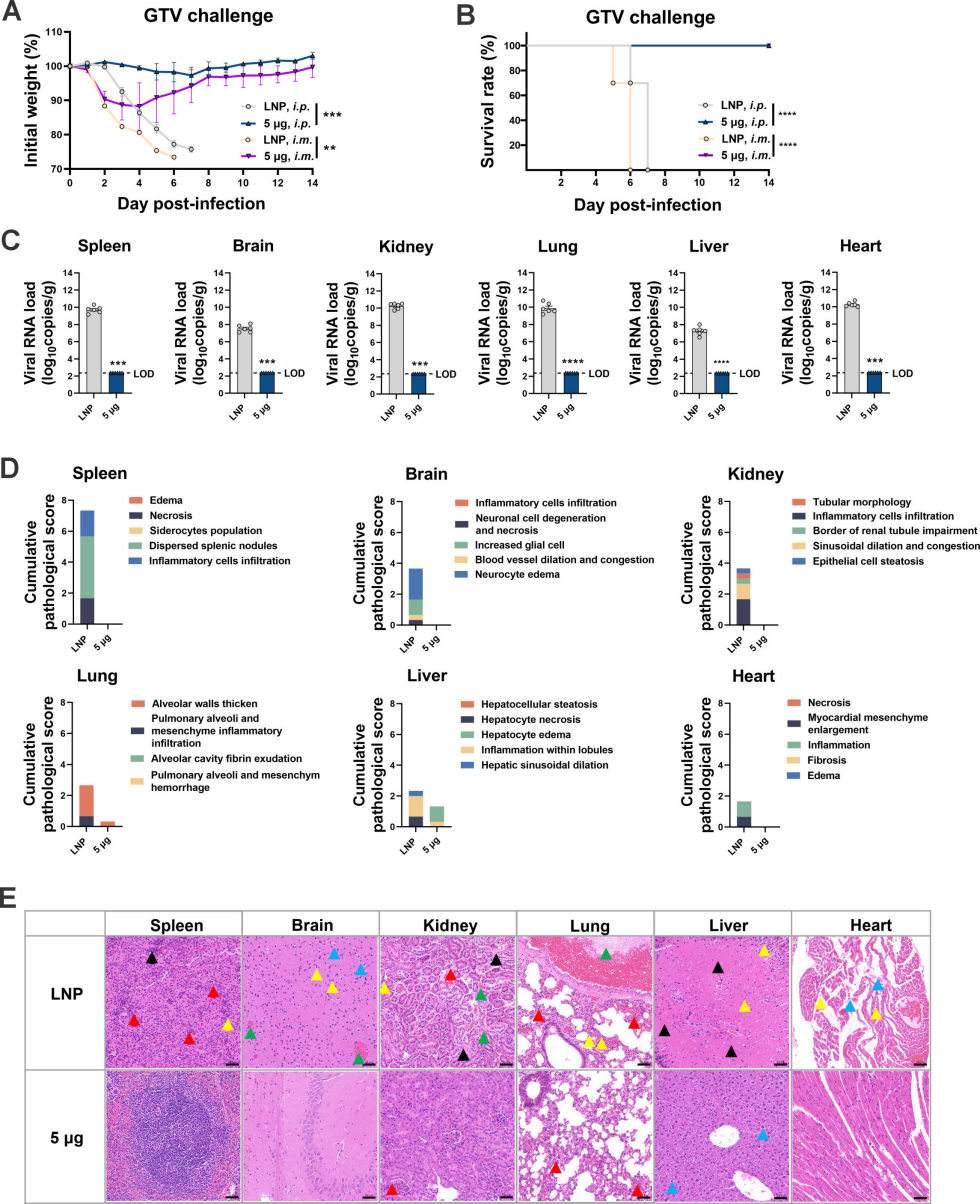
Cross-protection of the *GP* mRNA vaccine against Guertu virus. A129 mice were vaccinated with empty LNPs or 5 µg of GP-LNPs using a two-shot regimen and were subsequently challenged with GTV (i.p., 100 TCID_50_; i.m., 1 × 10^5^ TCID_50_). (A and B) Body weight and survival were monitored for 14 days post-challenge. (C) Viral loads in tissues after i.p. challenge were quantified using reverse transcription-quantitative PCR, as described above (LOD, 232 copies). (D and E) Cumulative pathological scores and representative images from each tissue are presented. A detailed description of the pathological analysis is listed in the supplemental materials, and annotations for the colored arrows can be referred to in the Fig. 2 legend. **P < 0.01, ***P < 0.001, ****P < 0.0001. GTV, Guertu virus.

Based on the abovementioned results, the protective scope of the full-length SFTSV *GP* mRNA vaccine was investigated among bandaviruses, such as HRTV and GTV, which are evolutionarily close to SFTSV. Following a two-shot regimen with 5 µg of GP-LNPs, A129 mice and AG129 mice (IFNα/β/γR^−/−^) (32) were challenged with HRTV (A129, 1 × 10^7^ TCID_50_ i.p.; AG129, 1 × 10^5^ TCID_50_ i.m.) or GTV (A129, 100 TCID_50_ i.p.; A129, 1 × 10^5^ TCID_50_ i.m.). Unexpectedly, all GP-LNP-vaccinated mice survived from both HRTV and GTV challenge, regardless of the mouse background or challenge route (the HRTV data are shown in Fig. 5, and the GTV data are shown in Fig. 6). As exhibited in Fig. 5, mice in the vehicle group that received the i.m. challenge died later than those that received the i.p. challenge, yet the *GP* mRNA vaccine saved the mice from both challenge routes while maintaining their body weights. The viral loads and pathological data from the i.p. challenge group showed sterilizing viral clearance and obvious pathological elimination or alleviation in the spleens, livers, brains, lungs, hearts, and kidneys. As shown in Fig. 6, after receiving i.m. or i.p. challenge, all the mice in the vehicle group died at approximately 6 dpi, while the *GP* mRNA vaccine saved the mice from both challenge routes. Similarly, the viral loads and pathological lesions were thoroughly eliminated or significantly alleviated.

Despite cross-protection, no cross-binding or neutralizing antibodies were detected against HRTV or GTV (Fig. S4), suggesting that cross-cellular immunity might have occurred behind the protection. Collectively, these findings demonstrate the broader application scope of the full-length SFTSV *GP* mRNA vaccine and emphasize the crucial role of cellular immunity for cross-protection.

### Conserved epitopes within SFTSV GP among *Phenuiviridae* pathogens may contribute to cross-protection

**Fig 7:**
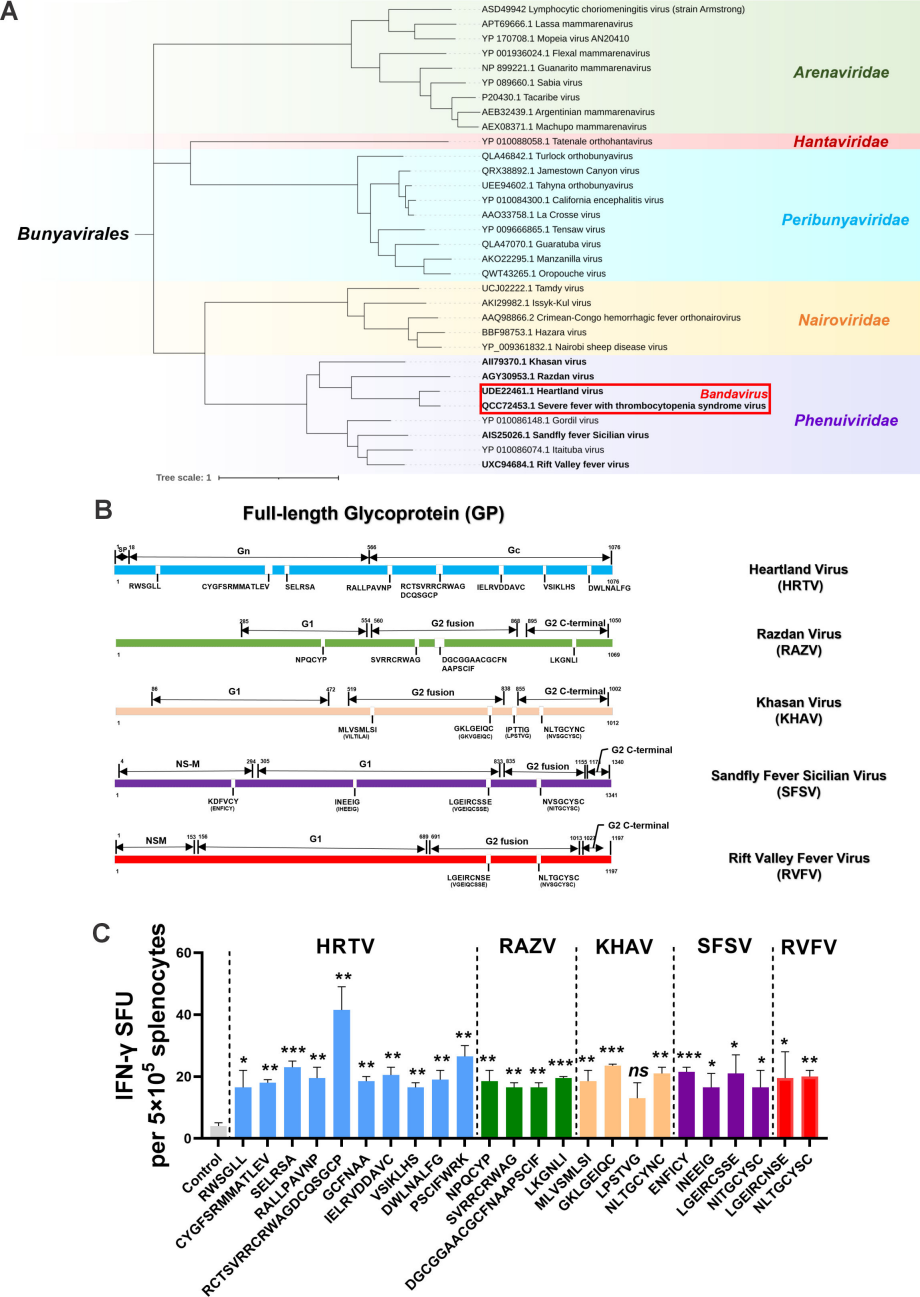
Identification of the potential protective epitopes from SFTSV GP. (A) Phylogenetic tree of the pathogens in the order *Bunyavirales* and pathogens from the genus *Bandavirus*, family Phenuiviridae, is highlighted. (B) Mapping of conserved and similar peptides from the SFTSV GP protein sequences among pathogenic Phenuiviridae pathogens. (C) IFN-γ secretion upon restimulation with each peptide (50 µg/mL) was detected by ELISpot, followed by statistical analyses comparing with the control. *P < 0.05, **P < 0.01, ***P < 0.001.

Inspired by the above results, GPs of *Phenuiviridae* pathogens, including HRTV, RAZV, KHAV, SFSV, and RVFV, were subjected to sequence alignment and T-cell epitope analysis, and a set of potentially conserved epitopes was founded in the SFTSV GP, particularly in the Gc domain. Among the five pathogens, HRTV had the most conserved epitopes with SFTSV, which was consistent with their evolutionary distance (Fig. 7A and B). Using an ELISpot assay, we used 24 peptides independently for *in vitro* stimulation of splenocytes from mice vaccinated with the SFTSV *GP* mRNA vaccine. As shown in Fig. 7C, all but one of the peptides induced significantly IFN-γ release. This experiment identified a set of potential protective T-cell epitopes within the SFTSV GP while also revealing the probable underlying mechanism of the observed cross-protection, suggesting the possibility of developing a broad-spectrum vaccine against *Phenuiviridae* pathogens based on conserved epitopes and antigens containing these epitopes.

## DISCUSSION

Pathogens carried by vectors, such as mosquitoes, ticks, sandflies, and midges, pose a great threat to human health when they spill over into humans, particularly during outdoor activities. Members of the *Phenuiviridae* family are globally prevalent, with a heightened incidence in underdeveloped regions, yet few vaccines or therapeutics are available. Given the similarities in terms of infection mechanism, transmission route, and virological and genetic properties, there is both a need and the potential for the development of broad-spectrum vaccines to combat these pathogens.

In this study, we present a candidate mRNA vaccine that employs the complete sequence of the SFTSV GP, leaving the secretory signal peptides unaltered and retaining the transmembrane and intracellular domains, thus ensuring its *in situ* expression and processing in a manner as natural as possible. This approach resulted in good efficacy not only against SFTSV but also against related bandaviruses, such as HRTV and GTV, exhibiting serendipitous effects despite seemly being unsophisticated. The glycoproteins of bandaviruses and *Phenuiviridae* viruses typically consist of a loose complex of subunits with irregular distribution on the viral membrane surface, which complicates their reproduction in an authentic conformation, posing a considerable challenge to recombinant protein vaccine development (19). Previous attempts to generate vaccines or therapeutic antibodies using recombinant Gn proteins have not yielded satisfactory results (33–35). Hence, preserving the active conformation while simultaneously inducing a robust cellular immune response is critical for a promising bandavirus vaccine, a view that is widely accepted among vaccine researchers. Thus, the use of mRNA technology to express the full-length glycoprotein *in situ* is necessary and feasible to some extent.

Consistent with our findings, Kim et al. reported that an mRNA vaccine basing the Gn head domain of SFTSV that elicits a robust humoral immune response and confers complete protection against SFTSV infection (27). Kwak et al. described the effectiveness of a DNA vaccine encoding Gn and Gc using a eukaryotic expression plasmid that successfully shielded ferrets from lethal challenge (36). These findings, along with the results of our study, corroborate the efficacy of nucleic acid vaccines derived from the SFTSV GP. Notably, our research illuminates the broad-spectrum potential of a full-length *GP* mRNA vaccine against bandaviruses and identifies protective epitopes on SFTSV GP. Moreover, our findings suggested that complete protection could be achieved by full-length GP-induced humoral and Th1-biased cellular immune responses or, in certain cases, solely by cellular responses. This outcome somewhat differs from those of previous studies, which reported that only a humoral response elicited by Gn/Gc or only a cellular response prompted by a combination of NP, NS, and LP can offer complete protection. The discrepancies between these studies may be attributed to differences in vaccine reactivity among species and individuals.

However, Kwak’s study and ours have jointly proposed an approach to develop an SFTSV vaccine inducing cellular immunity, which can be strongly triggered by nucleic acid vaccines, whether or not the antigens are from the receptor-binding domain. This concept can be employed in the development of broad-spectrum vaccines if conserved antigens are not limited to the receptor-binding domain or envelope glycoproteins. This idea was previously validated in a SARS-CoV-2 study (37). Zhong et al. reported the efficacy of a multigenic SARS-CoV-2 vaccine, which contained both spike and nucleoprotein genes, that significantly reduced viral loads and inflammatory cytokine production even in the absence of neutralizing antibodies (38). In addition, various approaches for developing broad-spectrum vaccines, such as using combinations of multiple antigens (39), employing sequential immunizations with different antigens (40), and incorporating combinations of conserved antigens or epitopes (41), have been proposed. Despite those, this study underscores the importance of utilizing antigens with as many conserved T-cell epitopes as possible.

Overall, although our study demonstrates the effectiveness of full-length SFTSV *GP* mRNA at providing broad-spectrum protection and reveals the potential role of conserved T-cell epitopes, cautions should be taken using large proteins as antigens, since the present technical bottleneck in preparing oversized mRNAs and the possible presence of adverse epitopes that may influence the final outcomes. In addition, the use of animal models for efficacy evaluations in preclinical studies may lead to deviations in protection, especially for bandaviruses like SFTSV, which lack both immunocompetent and low-cost rodent models (42, 43). Therefore, it seems necessary to evaluate the efficacy of this candidate vaccine in additional animal models before proceeding to clinical trials and clinical application.

## MATERIALS AND METHODS

### Proteins, antibodies, cell lines, and viruses

The ectodomains of SFTSV Gn (20–452 aa) and Gc (563–1,035 aa), GP (a heterodimer of ecto-Gn and Gc), and NP were recombinantly expressed in Expi293F cells, and subsequently purified via affinity chromatography. Polyclonal antibodies against SFTSV NP were prepared by immunization of New Zealand white rabbits.

Vero-ATCC and HEK-293T cells were cultured in Dulbecco’s modified Eagle’s medium (DMEM; Gibco, Grand Island, NY, USA) supplemented with 10% fetal bovine serum (FBS, Gibco) and maintained at 37°C with 5% CO_2_.

Authentic SFTSV (HBMC16 strain), HRTV (MO-4 strain), and GTV (DXM strain) specimens were preserved and obtained from the National Virus Resource Center, Wuhan Institute of Virology, Chinese Academy of Sciences, and propagated with Vero-ATCC cells. Animal experiments involving SFTSV and HRTV were conducted in an ABSL-3 laboratory.

### Peptides and peptide pools

The SFTSV GP sequences were uploaded to the Immune Epitope Database (https://www.iedb.org/) to predict major histocompatibility complex class I and II binding epitopes, after which the top 10 epitopes from either Gn or Gc were chosen for synthesis (GenScript, Nanjing, China). The peptides were dissolved in dimethyl sulfoxide and phosphate-buffered saline (PBS) and then mixed to form the SFTSV GP peptide pool.

The conserved or similar regions of HRTV, RAZV, KHAV, SFSV, and RVFV within SFTSV GP were identified as follows. First, the amino acid sequences of the HRTV, RAZV, KHAV, SFSV, and RVFV GPs were aligned with the SFTSV GP sequence using a local alignment method (Smith-Waterman) and conserved, or similar peptides containing six or more amino acids were selected for further analysis. Next, these conserved or similar peptides were evaluated for their T-cell immunogenicity using VaxiJen v.2.0 (44) and IFNepitope (https://webs.iiitd.edu.in/raghava/ifnepitope/application.php). Ultimately, 10 peptides were selected from HRTV; 4 peptides were selected from RAZV, KHAV, or SFSV; and 2 peptides were selected from RVFV.

### Generation and characterization of the *GP*-mRNA-LNPs

A full-length GP mRNA vaccine was developed based on SFTSV strain HB29 (GenBank: YP_006504094.1). The mRNAs and LNPs were produced using the Liverna Therapeutics platform (China patent: ZL201911042634.2). Briefly, mRNA was synthesized using an optimized T7 RNA polymerase-mediated transcription reaction *in vitro* with the complete replacement of uridine with N1-methyl-pseudouridine. The reaction was performed using a DNA template bearing an open reading frame bordered by 5′ and 3′ untranslated regions, and concluded with the incorporation of a poly A tail. The length and purity of the *in vitro*-transcribed mRNAs were further validated through microchip capillary electrophoresis (5200 Fragment Analyzer system; Agilent, Santa Clara, USA), and the mRNAs were then encapsulated in LNPs according to a refined procedure, wherein an ethanolic lipid mixture of ionizable cationic lipids, phosphatidylcholine, cholesterol, and polyethylene glycol lipids was rapidly mixed with an aqueous solution containing the mRNA products. Subsequent analytical assessments involved measuring the particle size, polydispersity index, encapsulation efficiency, pH, endotoxin contamination, and bioburden.

### Animal immunization and challenge

BALB/c mice (aged 6–8 weeks, *n* = 5) and A129 or AG129 mice (aged 6–10 weeks, *n* = 6–10) received intramuscular vaccinations according to a prime-boost regimen at 3-week intervals. Serum samples were collected biweekly until sacrifice or challenge. Ten days after the booster vaccination, the mice were i.p. inoculated with 100 TCID_50_ of SFTSV (A129), 1 × 10^7^ TCID_50_ of HRTV (A129), or 100 TCID_50_ of GTV (A129), or i.m. inoculated with 1 × 10^5^ TCID_50_ of SFTSV (A129), HRTV (AG129), and GTV (A129). Body weights and survival were recorded daily from day 0 to 14. At the experimental endpoint, the spleens, livers, lungs, brains, kidneys, and hearts were dissected for viral detection and histopathological examination.

### Enzyme-linked immunosorbent assay

Antibody titers specific to SFTSV Gn, Gc, or GP were determined using classical enzyme-linked immunosorbent assays. Briefly, the Gn, Gc, and GP proteins were diluted to 2 µg/mL in coating buffer (0.1-M sodium carbonate, 0.1-M sodium bicarbonate, pH 9.6), and then dispensed into each well of a 96-well polystyrene high-binding flat-bottom plate (Greiner, Frickenhausen, Germany) at 100 µL/well, followed by incubation at 4°C overnight. The next day, the plates were washed three times with PBS with 0.05% Tween 20 (PBST) and then incubated with blocking buffer (PBST, 5% skim milk) for 1 hour. Serially diluted sera (beginning with a 1:100 dilution) samples were added to the wells and incubated at room temperature for 1 hour. After further washing, the wells were incubated with horseradish peroxidase-conjugated goat anti-mouse IgG (H + L) (ABclonal, Wuhan, China) at a dilution of 1:4,000 for 1 hour. After incubation, a tetramethylbenzidine solution (Proteintech, Rosemont, IL, USA) was added to each well, and the reaction was stopped with 2 M H_2_SO_4_ after 10 minutes. Finally, the absorbance was measured at 450 nm using a Synergy H1 microplate reader (BioTek, Winooski, VT, USA). Absorbance values greater than twice that of the control (PBS) were considered positive.

### ELISpot assays

Splenocytes from vaccinated mice were processed through a 70-µm mesh filter, followed by suspension in RBC lysis buffer (Cell Signaling Technology, Danvers, MA, USA) for 10 minutes at room temperature, shielded from light. Subsequently, 1 × 10^6^ cells were cultured with the SFTSV GP pool (2 µg/mL each peptide) in ELISpot plates (MabTech, Stockholm, Sweden) at 37°C for 36 hours, employing phorbol 12-myristate 13-acetate/ionomycin and RPMI 1640 medium as positive and negative controls, respectively. After incubation, spots indicative of IFN-γ, IL-2, IL-4, and IL-10 secretion were detected as per the ELISpot Plus assay protocol (MabTech) and enumerated using an ImmunoSpot S6 plate reader (Cellular Technology Limited, Shaker Heights, OH, USA).

To identify conserved peptides, splenocytes from vaccinated mice were cultured in ELISpot plates at a density of 5 × 10^5^ cells per well. The cells were then stimulated with each peptide (50 µg/mL) derived from HRTV, RAZV, KHAV, SFSV, and RVFV individually for 36 hours at 37°C. The subsequent procedures were performed according to the methodology described above.

### Intracellular cytokine staining assay and flow cytometry

Splenocytes were stimulated with the SFTSV GP peptide pool (2 µg/mL each peptide) supplemented with 5-µg/mL brefeldin A (Absin, Shanghai, China), followed by a 10-hour incubation prior to cell labeling. First, live and dead cells were distinguished using the Zombie Aqua Fixable Viability Kit (BioLegend, San Diego, CA, USA). Then, the Fc receptors were blocked with purified rat anti-mouse CD16/CD32 antibodies (clone 2.4G2; BD Pharmingen, San Diego, CA, USA). Next, primary antibodies, including anti-CD3e-APC-Cy7 (clone 145–2C11), anti-CD4-PE (clone RM4-5), anti-CD8a-PE-Cy7 (clone 53–6.7), and anti-CD44-APC (clone IM7), all from BD Pharmingen, were added to the cells and incubated at 4°C for 30 minutes. Following cell surface labeling, the splenocytes were fixed and permeabilized using a fixation/permeabilization kit (BD Pharmingen) before the addition of anti-IFN-γ-BV786 (clone XMG1.2) and anti-IL-4-BV711 (clone 11B11) antibodies. The data were collected using a BD LSRFortessa instrument (BD Biosciences, Franklin Lakes, NJ, USA), and subsequent analysis was conducted using FlowJo v.10 (BD Biosciences).

### Plaque reduction neutralization test (PRNT)

Vero-ATCC cells were seeded into 48-well plates at a density of 2 × 10^5^ cells/well and cultured overnight. The next day, serially diluted sera (beginning with a 1:15 dilution) in DMEM containing 2% FBS were incubated with 200 plaque-forming units of SFTSV, HRTV, or GTV at 37°C for 1 hour. The serum-virus mixture was then added to duplicate wells and incubated with the cells for an additional hour. After incubation, the mixture was discarded, and the cell monolayer was washed with PBS and overlaid with 1.25% methylcellulose and DMEM containing 2% FBS. Three days later, the overlay was removed and the cells were fixed in 4% formaldehyde and incubated with 0.2% Triton X-100 in 5% skim milk for 30 minutes. Immunoplaques were detected using laboratory-prepared anti-SFTSV NP rabbit polyclonal antibodies (1:1,000), horseradish peroxidase (HRP)-conjugated goat anti-rabbit IgG (1:1,000) (ABclonal), and an Enhanced HRP-DAB Chromogenic Kit (TIANGEN, Beijing, China). The spots were recorded using an ImmunoSpot S6 reader (Cellular Technology Limited), and PRNT_50_ and GMT values were calculated using GraphPad Prism v.9 (GraphPad, San Diego, CA, USA).

### Quantification of viral copies in tissues

Total RNA was extracted from spleens, livers, lungs, brains, kidneys, and hearts using the RNeasy Mini Kit (Qiagen, Hilden, Germany) according to the manufacturer’s instructions. Subsequently, 3 µL of RNA from a 30-µL elution was reverse transcribed using the HiScript III All-In-One RT SuperMix Perfect for quantitative PCR (qPCR) kit (Vazyme, Nanjing, China) in a reaction volume of 20 µL. Then, 2 µL of cDNA was amplified by qPCR utilizing the Taq Pro Universal SYBR qPCR Master Mix kit (Vazyme), with primers specific for the *NP* genes of SFTSV, HRTV, or GTV. Amplification was conducted using a QuantStudio v.6 Pro thermocycler (ABI, Natick, MA, USA), and viral copies were quantified using a standard curve generated from plasmids encoding NP.

### Histopathology

Tissues dissected from animals were immediately fixed in 4% paraformaldehyde. The fixed tissues were subsequently dehydrated with an ethanol gradient and embedded in paraffin. Sections (4 µm thick) were cut from the embedded tissues, dried at 60°C, then dewaxed and stained with hematoxylin and eosin. The stained sections were dehydrated, sealed, and imaged using a Nikon Digital Sight DS-FI2 microscopic imaging system (Nikon, Tokyo, Japan). Pathological scoring was performed by a professional animal pathology analysis agency (Wuhan BaiQianDu Biotechnology Co., Ltd., Wuhan, China) using objective scoring criteria (see supplemental materials).

### Data analysis

Data were processed using GraphPad Prism v.9.0 software, and the results are displayed as the mean ± standard error of the mean. Statistical analyses comprised both unpaired Student’s *t*-tests and Mann-Whitney *U* tests, with significance denoted as not significant (ns) for *P* > 0.05, * for *P* < 0.05, ** for *P* < 0.01, *** for *P* < 0.001, and **** for *P* < 0.0001.

## Data Availability

All data associated with this study are included in the main text and supplemental material. Inquiries regarding materials, data, and the elaboration of methods should be addressed to the primary contact, Xiaoyan Pan (panxy@wh.iov.cn).

## SUPPLEMENTAL MATERIAL

Supplemental material jvi.00769-24-s0001.docx Figures S1 to S4; detailed pathological scoring criteria for each kind of tissue.

ASM does not own the copyrights to Supplemental Material that may be linked to, or accessed through, an article. The authors have granted ASM a non-exclusive, world-wide license to publish the Supplemental Material files. Please contact the corresponding author directly for reuse.
