# Supplementary material for: A full-length glycoprotein mRNA vaccine confers complete protection against severe fever with thrombocytopenia syndrome virus, with broad-spectrum protective effects against bandaviruses
Source: J Virol. 2024 Jun 3;98(7):e00769-24. doi: 10.1128/jvi.00769-24 (PMC11265342; doi:10.1128/jvi.00769-24)
Supplement: Supplemental material — Figures S1 to S4; detailed pathological scoring criteria for each kind of tissue. [file jvi.00769-24-s0001.docx]

# Supplementary materials

Figure S1 Analysis of SFTSV *GP* mRNA by microchip capillary electrophoresis via Agilent 5200 Fragment Analyzer system.

The size of the full-length SFTSV *GP* mRNA is 3490 nt, the purity is 98.3%.

Figure S2 Detection of the full-length SFTSV *GP* mRNA expression in mammalian cells.

The expression of SFTSV *GP* mRNA vaccine was conducted in HEK-293T cells by Western blot, GAPDH was made as a control.

(on same membrane)

**Anti-Gn (1:4000)**

**M**


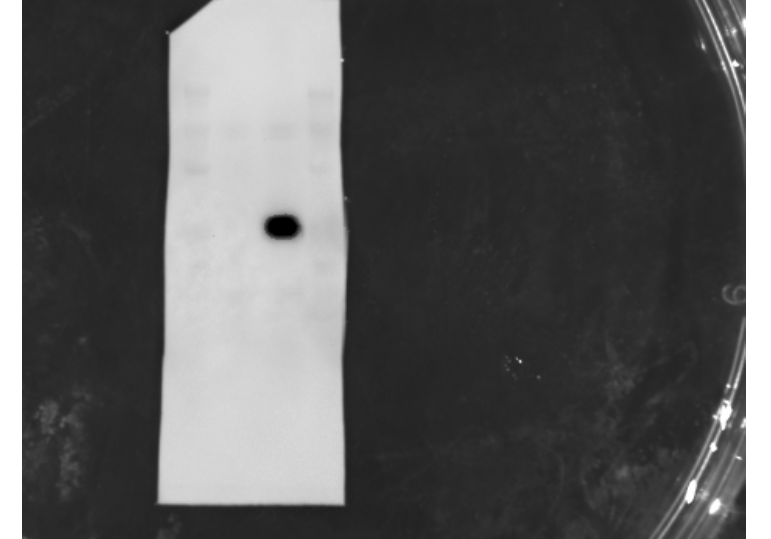


**GP**

**LNP**

**GP-LNP**

**GAPDH**


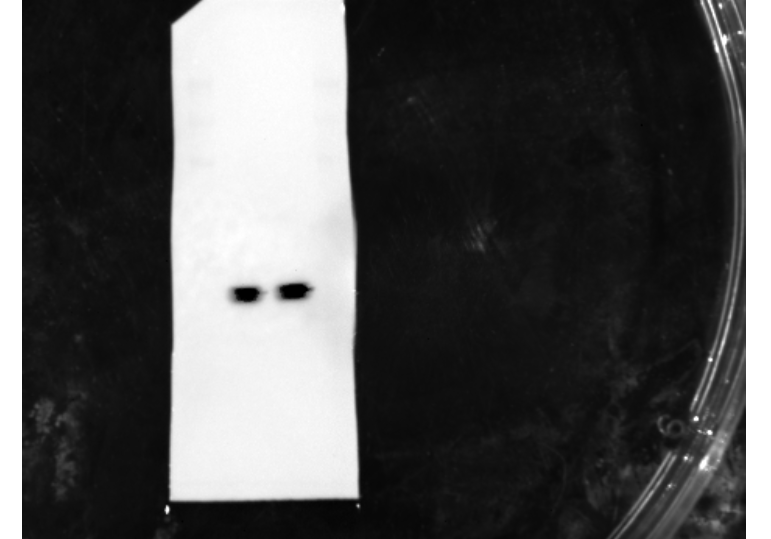


**GP-LNP**

**LNP**

**M**

**Anti-GAPDH (1:4000)**

**Figure S3 Detection of the cellular immune response triggered by the SFTSV *GP* mRNA vaccine.**

Splenocytes from the SFTSV *GP* mRNA vaccine-vaccinated mice were isolated for *in vitro* stimulation with the SFTSV GP peptide pool, and then detected for immune cell marker and intracellular cytokines expression by flow cytometry followed by cell staining. Representative pictures for Th1 (IFN-γ^+^, CD4^+^, CD3^+^), Th2 (IL-4^+^, CD4^+^, CD3^+^), CTL (IFN-γ^+^, CD8^+^, CD3^+^), and Tem (IFN-γ^+^, CD44^+^, CD8^+^, CD3^+^) cell populations were presented.

**Figure S4** **The cross- neutralizing and binding antibodies against HRTV and GTV**.

**B**

**(Sera from AG129)**

**(Sera from A129)**

**A**


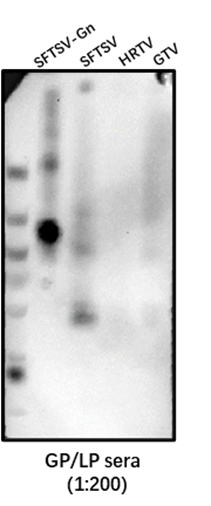

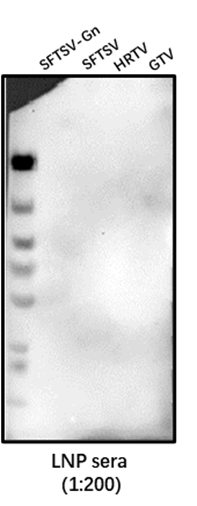


**C**

The neutralizing titer of SFTSV *GP* mRNA-vaccinated mice was detected by PRNT against HRTV or GTV (**A** and **B**), while none cross-neutralizing antibodies could be detected. The binding antibodies was detected against SFTSV-, HRTV- or GTV- infected Vero-ATCC cells by Western blot (**C**). SFTSV recombinant Gn protein was made as a positive control of antigen. None cross-binding antibodies could be detected.

## Detailed pathological scoring criteria for each kind of tissue

1. **Lung**
2. Pulmonary alveoli and mesenchyme inflammatory infiltration:

0’’: without inflammatory infiltration;

1”: infiltration with only several inflammatory cells;

2”: infiltration with a small amount of inflammatory cells;

3”: infiltration with a mass of inflammatory cells, or forming focal inflammatory.

1. Alveolar walls thicken:

0”: without thickening;

1”: thickening in mild;

2”: thickening obviously;

3”: lose normal alveolus pulmonis structure.

1. Pulmonary alveoli and mesenchym hemorrhage:

0’’: no erythrocyte in lumen;

1”: a small amount of erythrocyte in lumen;

2”: a mass of erythrocyte in lumen;

3”: the lumen was filled with erythrocyte.

1. Alveolar cavity fibrin exudation:

0”: without exudation;

1”: exudation in low level;

2”: exudation in high level;

3”: the alveolar space was almost filled with exudation.

1. **Liver**
2. Hepatocyte necrosis:

0’’: without necrosis

1”: the area of necrosis was less than 10 %;

2”: the area of necrosis was between 11% to 50%;

3”: the area of necrosis was between 51% to 75%;

4”: the area of necrosis was greater than 75%.

1. Hepatocellular steatosis:

0’’: the degree of steatosis less than 5%;

1”: the degree of steatosis was between 5%-33%;

2”: the degree of steatosis was between 34%-66%;

3”: the degree of steatosis was more than 66%.

1. Inflammation within lobules:

0”: no lobules contain inflammation;

1”: the quantity of lobules contain inflammation was less than 2;

2”: the quantity of lobules contain inflammation was between 2-4;

3”: the quantity of lobules contain inflammation was more than 4.

1. Hepatocyte edema:

0”: without edema;

1”: the level of edema was low;

2”: the level of edema was moderate or severe.

1. Hepatic sinusoidal dilation:

0”: without dilation;

1”: the level of dilation was low;

2”: the level of dilation was severe.

1. **Brain**

0”: without lesion

1”: minimal lesion

2”: slight lesion

3”: moderate lesion

4”: severe lesion

1. **Spleen**
2. Necrosis:

0”: without necrosis;

1”: the area of necrosis was less than 10%;

2”: the area of necrosis was between 11%-50%;

3”: the area of necrosis was between 51%-75%;

4”: the area of necrosis was more than 75%.

1. Edema:

0”: the area of edema was less than 2%;

1”: the area of edema was between 2%-15%;

2”: the area of edema was between 16%-45%;

3”: the area of edema was between 46%-75%;

4”: the area of edema was more than 75%.

1. Siderocytes population (the quantity of siderocytes in 200X field):

0”: less than 10;

1”: between 11-50;

2”: between 51-90;

3”: between 91-130;

4”: more than 130.

1. Dispersed splenic nodules:

0”: the morphology of splenic nodules was normal;

1”: 1-2 of splenic nodules appeared irregular morphology;

2”: lots of splenic nodules appeared irregular morphology, and obscure boundary;

3”: most of splenic nodules appeared irregular morphology, and obscure boundary;

4”: lacking splenic nodules structure, small lymphocytes aggregating.

1. Inflammatory cells infiltration:

0”: without inflammatory;

1”: slight infiltration;

2”: moderate infiltration;

1. **Kidney**
2. Inflammatory cells infiltration:

0”: without inflammatory cells;

1”: the focus of inflammatory cell was less than 2;

2”: the focus of inflammatory cell was between 2-5;

3”: the infiltration area was between 30%-65% of tissue;

4”: the infiltration area was exceeded 65%.

1. Tubular morphology:

0”: without;

1”: less than 15%;

2”: between 16%-45%;

3”: between 46%-75%;

4”: more than 75%.

1. Sinusoidal dilation and congestion:

0”: less than 5%;

1”: between 5%-25%;

2”: between 26%-50%;

3”: between 51%-75%;

4”: more than 75%.

1. Border of renal tubule impairment:

0”: without;

1”: less than 5%;

2”: between 6%-25%;

3”: between 26%-65%;

4”: more than 65%.

1. Epithelial cell steatosis:

0”: less than 5%;

1”: between 5%-33%;

2”: between 34%-66%;

3”: more than 66%.

1. **Heart**
2. Myocardial mesenchyme enlargement:

0”: myocardial mesenchyme without enlargement;

1”: myocardial mesenchyme partially enlarged;

2”: myocardium separated partially or integrally, the interval of muscle bundle enlarged obviously;

3”: the interval of cardiac muscle fibers enlarged significantly, muscle bundle was separated.

1. Necrosis:

0”: without necrosis;

1”: the area of necrosis was less than 10%;

2”: the area of necrosis was between 11%-50%;

3”: the area of necrosis was between 51%-75%;

4”: the area of necrosis was more than 75%.

1. Fibrosis:

0”: without fibrous tissue hyperplasia;

1”: the area of fibrous tissue hyperplasia was less than 10%;

2”: the area of fibrous tissue hyperplasia was between 11%-50%;

3”: the area of fibrous tissue hyperplasia was between 51%-75%;

4”: the area of fibrous tissue hyperplasia was more than 75%.

1. Inflammation:

0”: without inflammation cells;

1”: the focus of inflammatory cell was less than 2;

2”: the focus of inflammatory cell was between 2-5;

3”: the infiltration area was between 30%-65% of tissue;

4”: the infiltration area was exceeded 65%.

1. Edema:

0”: less than 2%;

1”: between 2%-15%;

2”: between 16%-45%;

3”: between 46%-75%;

4”: more than 75%.
